# Optimization of Protein Extraction of Oenological Interest from Grape Seed Meal Using Design of Experiments and Response Surface Methodology

**DOI:** 10.3390/foods10010079

**Published:** 2021-01-04

**Authors:** Berta Baca-Bocanegra, Julio Nogales-Bueno, José Miguel Hernández-Hierro, Francisco José Heredia

**Affiliations:** 1 Food Colour and Quality Laboratory, Área de Nutrición y Bromatología, Facultad de Farmacia, Universidad de Sevilla, 41012 Sevilla, Spain; bbaca1@us.es (B.B.-B.); julionogales@us.es (J.N.-B.); heredia@us.es (F.J.H.); 2 Department of Animal Production, Campus de Rabanales, Universidad de Cordoba, 14071 Córdoba, Spain

**Keywords:** response surface methodology, optimization, grape seed, protein extraction, wine

## Abstract

After grape processing, large amounts of seeds are produced as a side product. Grape seeds are rich in proteins that could be extracted and revalorized by the wine industry due to their high techno-functional value for modulating quality features of red wines or as an alternative to the use of other stabilizers, which are also proteins but submitted to legal restrictions. Box–Behnken design and response surface methodology were used to optimize the protein extraction from defatted grape seed meal to maximize the protein purity of the isolate under practical operating conditions of pH, temperature, meal/water ratio and extraction time. The most significant factor was pH, both in the linear and quadratic forms. Additionally, the interaction between pH and temperature exhibited a significant effect on protein content. The removal of phenolic compounds from grapeseed meal, before optimizing protein extraction, led to a significant increase of 7.70% in the protein purity of grape seed protein concentrate. Therefore, grape seeds can be used as a sustainable way for protein production in the oenological sector due to the availability of grape seeds, their low cost as a grape-processing by-product, and the protein purity reached thanks to the described procedure for optimization of protein extraction.

## 1. Introduction

Food processing generates high amounts of residues which in most cases are directly eliminated as scrap products. Nowadays, there is an increasing demand for a sustainable food industry reducing the contamination effects of their wastes and increasing their value by transforming them into useful by-products or as raw material for other industries. Concerning the wine industry in particular, the important environmental impact of the residues obtained from grape vinification has been reported [1]. Grape pomace, comprised of the remains of seeds, skins, stems and pulp, constitutes one of the main wastes generated by winemaking procedures [2]. It is estimated that the aforesaid by-products represent approximately 20% of the grapes, by weight, used in the vinification procedure [3].

Different alternatives have already been carried out to revalorize grape pomace for several decades. Traditionally, grape pomace has been used to produce wine alcohol and has been used to extract the oil from grape seeds [2,4]. Other traditional applications of grape pomace have allowed its use as a fertilizer, fuel or animal feed [5]. In recent times, the most interesting approaches to revalorize grape pomace are aimed to develop useful products for the food industry since their components have been described as low-cost sources of many bioactive compounds [6].

Different studies focus on the composition of winemaking by-products have been reported. In general, grape pomace is a rich source of fiber, protein, cellulose, minerals and phenolic compounds [6]. The composition of wine pomace and then its main components could be notably affected by the composition of grapes and both the type of process and the conditions under which winemaking is carried out [7]. Many studies mainly focus on the polyphenols of grapes but not on the other components such as proteins. Grapes contain proteins in pulp, skin and seeds [8,9] and their protein profile varies with ripening [10] and environmental and stress stimuli [11,12]. There are different studies about the characterization of grapes skin and pulp proteins [10,12] but those that focus on seed proteins are scarcer. Wine proteins come mainly from the pulp and the skins since those of seeds do not transfer significantly. However, the characterization of grape seed protein acquires great importance since, after grape processing; large amounts of seeds are produced as a by-product. Taking that into account, they could be extracted and revalorized by the food industry and the wine industry in particular due to their high techno-functional value for modulating quality features such as the appearance, color and stability of red wines [13,14,15]. Moreover, grape seed proteins can represent a new alternative to the use of other stabilizers, also proteins, but submitted to legal restrictions. Inorganic materials and proteins of animal and vegetable origin have been used to perform wine clarification. In this sense, clarification studies carried out with vegetable proteins, showed a clarification capacity similar or superior to proteins of animal origin, with a generally smaller volume of lees [16,17]. Seed proteins have recently been proposed as clarifying agents [10,14,18]. Grape seed, therefore, may serve as a potential source of a rich functional protein isolate in the oenological sector.

In the agri-food sector, the protein extraction in alkaline solution followed by isoelectric precipitation is one of the most common methods reported in the literature [19,20,21]. The high solubility of proteins in alkaline conditions and their minimal solubility at their isoelectric point constitute the basis of this method. However, there are several factors such as pH, temperature, extracting time or the raw material–solvent ratio which could affect protein solubility and, therefore, protein extractability [22]. Taking that into account, it is interesting studying the procedure conditions that allow obtaining the best possible response, that is to say, the optimization of the protein isolate extraction.

The optimization procedure has been traditionally carried out by studying the influence of one factor maintaining the rest of the factors constant. However, in the majority of cases, the evaluated response is affected not only by a single factor but many factors and their interactions, as is the case of extraction of protein isolates according to their different pH-solubility. As it happens, response surface methodology (RSM) is a suitable tool for optimizing the analytical procedure [23]. Response surface methodology provides relevant information about a dataset in the shortest time with the least number of experiments [23]. Different studies have used RSM for the extraction optimization of proteins from different sources [24,25,26] and other compounds like phytochemicals [27] or flavanols and anthocyanins [28].

In this work, response surface methodology has been employed to optimize the protein extraction process from grape seeds meal for maximum protein purity in the protein isolate under the practical operating conditions of pH, temperature, meal/solvent ratio, and extraction time.

## 2. Materials and Methods

### 2.1. Grape Seeds Meal

Industrial defatted grape seed meal, provided by Alvinesa S. L. (Ciudad Real, Spain), was used in this work for protein extraction. At the Alvinesa facilities, grape pomace, produced as a by-product from the wine-making process of the grapes, is subjected to different processes to obtain some compounds of interest such as oil and polyphenols that are subsequently marketed. The residual grape seed flour is discarded and used as its fuel or fertilizer for the field. In detail, at the industrial plant, grape pomace was washed extensively with water, destemmed, and dried. During the washing process, a high proportion of the seed polyphenols and other compounds were eliminated. The dried grape seeds were separated from the rest of the grape pomace, ground and defatted using hexane. Then, the defatted grape seed meal was desolventized to remove the residual hexane. Finally, once it was received in our lab, the defatted grape seed meal was ground again in a laboratory mill to obtain the finest powder and then stored at room temperature in airtight containers for further analysis.

### 2.2. Proximate Composition of Defatted Grape Seeds Meal

The proximate composition of the defatted grape seed meal was analyzed. For it, protein content, fat, ash, moisture, and crude fiber were determined following the standard methods of the Association of Official Agricultural Chemists. All experiments were carried out in triplicate. The carbohydrate content was calculated as 100% minus the sum of the percentage of protein, moisture, fat, and ash contents.

### 2.3. Optimal pH of Protein Precipitation

Before the protein extraction, the optimal pH of grape seed protein precipitation was determined. For it, five identical alkaline suspensions of grape seeds meal were prepared (pH 10). After the solubilization of the proteins, the slurries were centrifuged at 14,000× *g* for 20 min and then, the supernatants were separated and the solubilized proteins in the supernatant from each solution were precipitated with HCl at a different pH level ranging from 3 to 4.6 (3, 3.4, 3.8, 4.2 and 4.6). Once the precipitate was removed by centrifugation in the aforementioned conditions, the absorbance at 280 nm of no precipitated protein was measured to determine the isoelectric point (pI) for the protein of grape seeds meal.

### 2.4. Protein Extraction and Optimization of Extraction Process: Response Surface Methodology (RSM)

Response surface methodology (RSM) was performed to estimate the effect of different variables on the protein purity of grape seed protein concentrate. After establishing the variables and preliminary range of these variables through the bibliography, a Box–Behnken Design (BBD) with four independent variables at three levels was performed. The studied variables and their levels were pH (x_1_) from 8.5 to 10.5, temperature (x_2_) from 25 to 45 °C, meal/water ratio (x_3_) from 6:1 to 12:1 (*v*/*w*) and extraction time (x_4_) from 1 to 3 h (Table 1). A total of 27 experiments were performed in triplicate and random order according to the BBD (Table 2).

The isolation of grape seed meal protein was carried through an alkaline extraction followed by isoelectric precipitation, one of the most common methods of protein extraction reported in the literature [14,20,24]. In detail, a defatted grape seed meal was extracted with deionized and alkalized water, with a particular meal/water ratio, constant agitation, and controlled temperature. KOH or HCl was used to keep the pH of the suspension constant during the extraction procedure. Once the extraction time considered in each experiment was concluded, the sludgy with the extracted protein was centrifuged at 14,000× *g* at 4 °C for 20 min and the supernatant was collected. The pH of the collected supernatant was adjusted at its isoelectric point with HCl and the solubilized proteins were precipitated and recovered by centrifugation at 14,000× *g* at 4 °C for 20 min followed by freeze-drying and storage in airtight containers for future analyses. From now on, we will refer to this product as GSPC (grape seed protein concentrate).

The GSPC protein content was calculated for all extraction experiments. For it, the nitrogen content of GSPC was determined using the Kjeldahl method. Protein content was obtained using the nitrogen-to-protein conversion factor of 5.75 and reported as the purity of grape seed protein concentrate.

Data from 27 extractions were set to a response surface design using a second-order polynomial. The protein purity of GSPC was selected as the response variable and an optimum protein extraction condition was selected, based on the maximum purity that could be raised from the defatted grape seed meal. Finally, the protein isolate from defatted grape seeds meal was extracted using the optimized variables and used in subsequent analysis. From now on, we will refer to this product as OGSPC (optimized grape seed protein concentrate).

The software for data processing Statistica Version 8.0 (StatSoft, Tulsa, OK, USA) was used for the experimental design and to analyze the experimental data.

### 2.5. Determination of Amino Acid Content of OGSPC

High-performance liquid chromatography with post-column derivatization was used to determine amino acid composition. For it, the optimized grape seed meal protein concentrate (OGSPC) was hydrolyzed using 6 M HCl at 120 °C for 24 h and then, the pH was adjusted to pH 2.2 with 10 M and 1 M NaOH. The adjusted sample was diluted with lithium citrate loading buffer (0.20 M, pH 2.2) after adding norleucine as internal standard. Before being analyzed by HPLC, the sample was filtered through a 0.22 μm filter (Millipore Millex-GV, Millipore, Burlington, MA, USA). A Biochrom 30+ Amino Acid Analyzer HPLC system (Biochrom, Cambridge, England) was equipped with a UV–Vis detector and high-pressure PEEK cation exchange column packed with Ultropac 8 cation exchange resin. Samples were analyzed in triplicate and the results were reported as an average.

### 2.6. Phenolic Compounds Extraction of Grape Seed Meal

One portion of defatted grape seed meal was subjected to a phenolic extraction before optimized protein extraction. Following the procedure described by Malik and Saini [29], the previously defatted meal was dispersed in methanol (60% *v*/*v*) for 2 h, with constant agitation at a meal to solvent ratio of 1:20 (*w*/*v*). Then, the obtained suspension was filtered, and the extraction procedure was repeated four times. Finally, the thus obtained defatted dephenolized meal was lyophilized. After that, a protein isolate was prepared from the portion of defatted meal free of polyphenols using the optimized method described above.

### 2.7. Demucilaging Temperature of Grape Seed Meal

Another portion of the defatted grape seed meal was demucilaged by soaking in Mili-Q water at a meal to water ratio of 1:18, at 60 °C, for 3 h following the method described by Kaushik et al. [30]. The process was repeated three times. After three extractions, the defatted demucilaged meal was lyophilized. After that, a protein isolate was prepared from the portion of the defatted meal free of mucilage using the optimized method previously described.

## 3. Results and Discussion

### 3.1. Proximate Composition of Grape Seed Meal

The proximate composition of defatted grape seed meal was 11.78% moisture, 2.87% ash, 22.86% fiber, 1.3% lipid, 10.76% protein (expressed as N × 5.75), and 50.43% carbohydrates by difference. Protein content, ash and moisture were nearly close to that found by Castriotta and Canella [31] and Fantozzi [32] in the same matrix; however, Felhi et al. [33] reported a lower protein content and moisture and higher ash than that obtained in this study. The observed differences could be attributed to the variability of the studied cultivars and climatic conditions. Moreover, the industrial origin of the grape seed meal used in this work and the different procedures to which it has been subjected may affect its composition. In this sense, the lipid content can only be compared with the results obtained by Castriotta and Canella [31] since only in this case the sample has also been previously defatted. Similar results have been obtained in both studies. Grape seed composition could also be compared with those of cereals and other oil seeds. Focusing on protein content, grape seed meal presented a protein percentage similar to that of the most common cereals although slightly less than the range of values described for the most common leguminous seeds [20].

### 3.2. Optimal pH of Protein Precipitation

**Figure 1:**
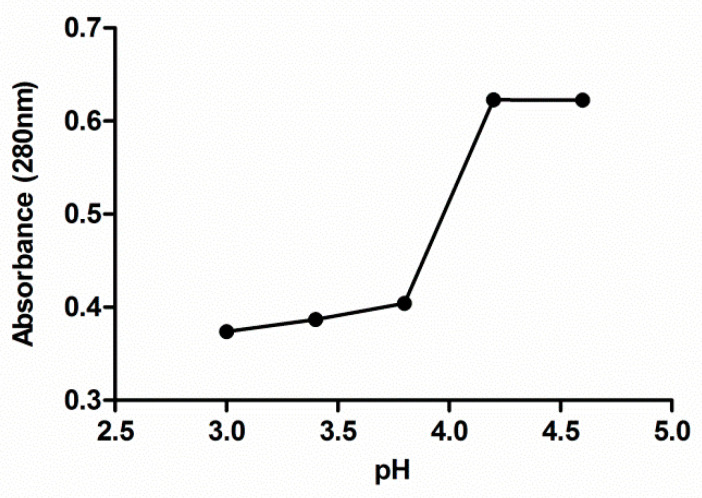
Effect of the pH on extracted grape seed meal protein precipitation. Each value is the mean of triplicate measurements.

The absorbance at 280 nm of no precipitated protein, remaining in the protein isolate supernatants after precipitation procedure, was plotted against the pH value used for the protein precipitation in each solution (Figure 1). A significant decrease in absorbance is observed from pH 4.2 obtaining the lowest absorbance value from pH 3.8. Below this value, the absorbance practically remains constant in the considered interval. Based on the data shown in Figure 1, pH 3.5 was selected in this study to separate seed proteins from the supernatant through isoelectric precipitation since it is the pH level with which it obtains less protein in the supernatant and therefore, more of them in the precipitate.

### 3.3. Optimization of Extraction Conditions by Response Surface Methodology (RSM)

#### 3.3.1. Response Surface Modeling

Grape seed meal was extracted for its protein following 27 combinations of four independent variables (pH, temperature, meal/water ratio and extraction time) as indicated by the experimental Box–Behnken design. The effects of the considered independent variables and their interactions on the protein content of grape seed protein concentrate are presented in Table 2. According to these data, the protein content of the GSPC ranged from 34.81 to 59.13 g protein/100 g of protein isolate under the tested conditions. Comparable results have been reported by Zhou et al. [15] (64.85%) and Gazzola et al. [14] (43.60%) in their isolated protein grape seed meal. The differences could be due to the different extraction processes or to the different values of the evaluated variables in the extraction process. It could not be excluded that the nature of grape seeds has also affected the protein content. As a general trend, the amount of protein extracted significantly increases with increasing pH up to 9.5, beyond this value the protein content decreases but not significantly. Other researchers have described a similar trend in protein extraction according to the pH. An increase in protein extraction with increasing pH extraction was also found for grape seed by Chenyan et al. [34]. In this work, the maximum protein extraction was reached at pH 9.5. As a consequence of the increase in pH, the negative charge of proteins increases and the electrostatic repulsion between them is enhanced. As a result, an increase in protein solubility takes place due to the protein–water interactions [35]. However, strong alkali conditions lead to protein denaturation, giving way to a decrease in protein extraction [22]. From the results shown in Table 2, it is not possible to infer the effect of the remaining factors on the response variable. It should be noted that the extraction could be affected not only by the single factor but also by the interactions between them.

Independent and dependent variables were fitted by a second-order polynomial equation to the experimental data. The regression equation obtained for protein content is shown below:

Y = −713.726 + 132.810 x_1_ − 12.887 x_2_ + 4.034 x_3_ + 907.545 x_4_ − 5.341 x_1_^2^ − 0.043 x_2_^2^ + 0.029 x_3_^2^ − 830.218 x_4_^2^ + 0.955 x_1 × 2_ − 0.592 x_1 × 3_ − 66.581 x_1 × 4_ + 0.038 x_2 × 3_ +20.290 x_2 × 4_ − 3.053 x_3 × 4_

where Y is the predicted response (protein content) and xi is the independent variable (pH (x_1_), temperature (x_2_), meal/water ratio (x_3_) and extraction time (x_4_)).

Using the second-order model, the respective predicted values of Y were obtained and compared with the experimental values (Table 2). The coefficient of correlation of the model (*R*^2^ = 0.80743) indicates a reasonable fit of the model to the experimental data. ANOVA was used as a mean to confirm the adequacy of the suggested model, verify the significance of each coefficient and evaluate the interaction strength of each parameter for the extraction procedure (Table 3). The lack of fit did not result in a significant *p*-value for the evaluated variables. So, this model fitted the data well and therefore it is sufficiently suitable for predicting the relevant response. In the present study, pH is the most relevant factor (*p* < 0.05), both in their linear and quadratic forms. Also, the results revealed that the interaction between pH and temperature exhibited a significant effect on protein extraction. The rest of the variables showed a low influence on the model (Table 3). The coefficient for the linear term pH in the model equation indicates that an increase in pH leads to a higher extraction of protein in the extraction procedure. The square term of pH negatively affects the protein extraction and resulted in a parabolic trend in protein content. This means that protein extraction is effective at a higher value of pH, however, but beyond a certain level, an inverse behavior is followed. Concerning the interaction terms, the corresponding coefficient of pH-T indicates that these factors modulate the response oppositely, that is, when pH and temperature are increased simultaneously, the individual effects of these parameters on protein extraction are canceled. A similar effect between pH and temperature on the protein extraction of watermelon and *Prosopis cineraria* (L.) Druce seeds was observed by Wani et al. [26] and Deepanshu et al. [24], respectively.

**Figure 2:**
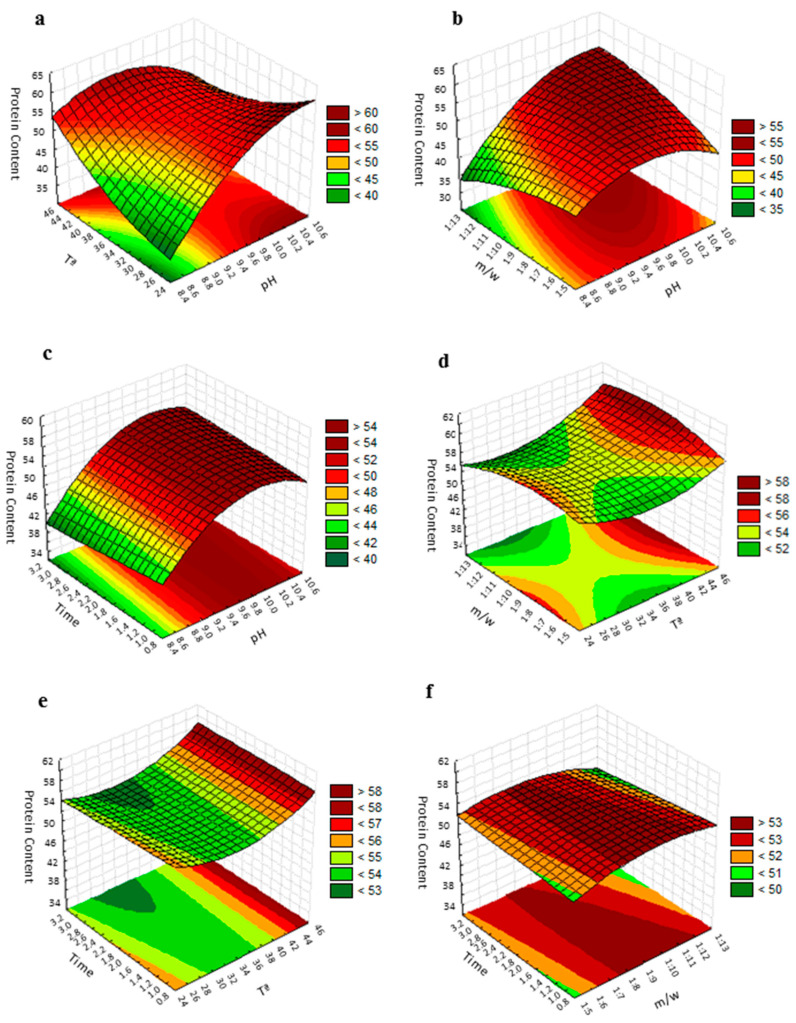
Response surface plot for the protein purity of the grape seed meal: (**a**) effect of pH and temperature on protein purity with the meal/water ratio of 1:9 and an extraction time of 2 h; (**b**) effect of pH and the meal/water ratio on protein purity with a temperature of 35 °C and an extraction time of 2 h; (**c**) effect of pH and extraction time on protein purity with the meal/water ratio of 1:9 and a temperature of 35 °C; (**d**) effect of temperature and the meal/water ratio on protein purity with a pH of 9.5 and an extraction time of 2 h; (**e**) effect of the temperature and extraction time on protein purity with a pH of 9.5 and a meal/water ratio of 1:9; (**f**) effect of the extraction time and the meal/water ratio on protein purity with a temperature of 35 °C and pH 9.5. Protein content is expressed as g protein/100 g CP, time in hours and T in °C.

The fitted polynomial equation was graphically represented as three-dimensional response surfaces to visualize the aforementioned relationship between the experimental levels of evaluated factors and response [36] (Figure 2). The figure shows the relative effects of two independent variables on protein content, while the other two factors were held at the zero coded level (center value of the testing ranges). The maximum protein content in the protein isolate was obtained when the pH was 10.5 and temperature was 25 °C, while the meal/water ratio and extraction time were kept at 1:9 (*w*/*v*) and 2 h, respectively (Figure 2a). An increase in pH significantly increased the amount of extracted protein; however, due to the influence of its square term, beyond a certain level an inverse trend was followed. Mizubuti et al. [37] and Deepanshu et al. [24] also reported the same quadratic effect in pigeon pea and *Prosopis cineraria* seeds protein extraction with pH. The inflection level is conditioned by the temperature. The elliptical shape of the contour plot, following the ANOVA of the model, indicates that the interaction between the pH and temperature are significant [38]. Variation in the pH and meal/water ratio revealed that protein purity was at its maximum when the pH was above 10.2 and the meal/water ratio above 1:9 (Figure 2b). As can be seen in the plot, the effect of the meal/water ratio factor could be considered practically irrelevant. In the same way, the variation of the extraction time did not show any significant effect on the protein content and then, the maximum protein content was obtained when the pH was above 10 for any time of extraction value evaluated in the study, when the temperature and meal/water ratio were fixed at their zero coded values (Figure 2c). Changes in the temperature and meal/water ratio revealed that the maximum protein content was obtained when the temperature was 45 °C and the meal/water ratio was 1:9 (Figure 2d). This maximum was maintained for a concentration range between 1:7 and 1:12, indicating that the variation in meal/water ratio did not show any profound effect on the protein extraction. As can be seen in Figure 2e, the maximum value of protein purity was obtained when the temperature was 45 °C for all evaluated extraction time values. As already mentioned, the extraction time did not affect protein extraction in the selected range. Finally, if how the purity of proteins is affected by the variations of extraction time and concentration (meal/water ratio) is evaluated, a representation like that of Figure 2f is obtained. The maximum protein content is reached in a concentration range between approximately 1:7.5 and 1:10 for all the extraction time values evaluated.

Considering the fitted equation and all the three-dimensional response surfaces, it is evident that the pH had a significant effect on protein extraction while the effect of temperature was more limited and it seems to be more related to its interaction with pH than to its effect. The extraction time and meal/solvent ratio did not seem to affect grape seed protein extraction in the selected range. It could be related to the long extraction time (1, 2 and 3 h) and small meal/solvent ratio (1:6, 1:9 and 1:12) evaluated in this study versus that used in other studies (extraction times ranged from 10 and 90 min and meal/solvent ratio between 1:20 and 1:80) in which these factors were significant for the response variable. Under these conditions, protein solubility could be compromised by the solubility equilibrium reached in lesser times and saturation effects produced by low amounts of solvent [34]. According to this reasoning, no significant effect was reported by Guerreo-Ochoa et al. [39] after evaluating the effect of 1 and 2 h of extraction time on quinoa (*Chenopodium quinoa* Wild.) protein extraction. In the same way, the meal/solvent ratio ranging between 1:5 and 1:25 showed no effect on pigeon pea protein extraction [37].

#### 3.3.2. Optimization Based on the Protein Content

Protein content and extraction yield are the responses most commonly used for the characterizations of protein isolates [24,25,40]. Due to the expected functional properties, related to its protein content, of the grape seed meal isolates in the oenological sector, the extraction optimization process based on protein purity was considered in this study. Consequently, the response surface methodology was applied to investigate the optimum conditions for maximizing the protein purity of protein concentrates obtained from grape seed meal.

The optimization procedure suggested that the extraction conditions of pH 9.94, the temperature of 36.42 °C, meal/water ratio 1:9.1 and extraction time of 2.19 h were the best conditions for the highest protein content of GSPC. To work under practical operating conditions, pH 10, 36 °C, 1:9 meal/solvent ratio and 2 h extraction time were considered. When these optimum values of independent variables were incorporated into the regression equation, 54.50 g protein/100 g of protein concentrate was obtained. To check the suitability of the model equation for predicting the optimum protein purity, the extraction was carried out, by three independent real experiments, at pH 10, the temperature of 36 °C, meal/solvent ratio of 1:9 and an extraction time of 2 h obtaining 55.35 g protein/100 g of OGSPC. The error between the predicted and experimental value of the protein content at optimized conditions was less than 2% which reveals the adequacy of the performed model. Taking that into account, pH 10, at 36 °C, the meal/water ratio of 1:9 and 2 h of extraction time were fixed as the optimized parameter values for protein extraction from grape seed meal.

Reported works about the optimization of the protein extraction from different protein sources showed a wide range of results. Studies focused on the extraction of grape seed meal protein are scarce; however, similar studies using other protein sources have been reported. Chenyan et al. [34] employed the RSM to optimize the protein extraction process also from grape seed but for a maximum yield, showing 35 °C, 1:22.5 (*w*/*v*), 9.8 and 29 min as optimum conditions. Values of pH and temperature were in agreement with the current study. A previous study on protein extraction from the tomato seed meal showed 50 °C, pH 11.5, 20 min and 1:30 as optimized values [41]. Firatligil et al. [25] reported a maximum protein extraction of red pepper seed using 31 °C, pH 8.8, 20 min and a 1:21 meal/water ratio. The highest extraction yield of grass pea protein was produced at pH 9.96, 1:15 (*w*/*v*) and with 58 min extraction time [40].

### 3.4. Amino Acid Composition of OGSPC

Amino acid composition of OGSPC is shown in Table 4. Glycine, aspartic acid, glutamic acid, and arginine were the most abundant amino acids found in OGSPC accounting for about 59% of the total amino acids. These results showed an amino acid profile of grape seeds similar to that of cereals and other oil seeds which are rich in glutamic acid and aspartic acid and deficient in tryptophan and sulfur-containing amino acids [42]. In the optimized protein concentrate obtained in this study from grape seed meal, tryptophan is not detected, while methionine and cysteine hardly represent 1.44 and 1% of the total amino acids, respectively.

Due to its similar composition to cereals, grape seeds and other oil seeds could be used as a substitute for these in foods. Obtaining enriched extracts of high interest for food applications is one of the most common approaches of the utilization and revalorization techniques of products and by-products of the agrofood industry due to both its nutritional and functional properties [15,25,30,42].

### 3.5. Polyphenol Removal and Demucilaging Temperature: Effect on the Purity of Protein Isolates

The optimization procedure carried out in this work allowed to reach an OGSPC protein content of 55.35%, a value which is higher than that reported previously by Gazzola, Vincenzi, Marangon, Pasini and Curioni [14] (43.6%) and Vincenzi, Dinnella, Recchia, Monteleone, Gazzola, Pasini and Curioni [18] (15.4%) for a similar extract and even using in their studies a higher nitrogen-to-protein conversion factor (6.25 vs. 5.75). Due to its protein content, OGSPC could represent a valuable fining agent for wine as an alternative to the traditionally used plant or animal exogenous protein. However, the protein content of the commercial fining agents used in vinification is higher than the obtained OGSPC. The low extraction yield in grape seed meal could be related to the existence of derived proteins or to the high content of fiber which prevents their extraction by blocking the process [31]. It is known that the interaction between proteins and polyphenols reduces the solubility profile of proteins [29]. To increase the protein content of the grape seed isolate, polyphenol removal and the demucilaging temperature of defatted grape seed meal was carried out independently before the protein extraction procedure. The obtained results are shown in Table 5. The removal of phenolic compounds from meal led to a significant (*p* < 0.05) increase of 7.70% in the protein content of OGSPC, reached 59.6 g protein/100 g of protein isolate, findings coincident with those of Malik et al. [29] in sunflower seeds. The removal of mucilage, before protein extraction, at the demucilaging temperature of 60 °C enhanced the protein content of grape seed protein concentrate from 55.35 to 58.06%, but in this case, the increase was not significant. Kaushik et al. [30] described a great improvement in the protein purity in flax seeds isolates due to the removal of mucilage using the same temperature. It could be taking into account that in this study an industrial grape seed meal has been used. Grape pomace, obtained as a by-product from the wine-making process of the grapes, was submitted to different procedures before obtaining the grape seed meal as it has been used. For example, it was washed extensively with water and then, a high proportion of polyphenols and especially mucilage could have been eliminated. It is logical that in samples in which these compounds have already been partially eliminated, the benefits of these interventions are more limited.

## 4. Conclusions

Response surface methodology and Box–Behnken design have been successfully used for the optimization of grape seed meal protein extraction to maximize the protein purity of the obtained protein isolate. According to the second-order model developed and the three-dimensional response surfaces, in the present study, pH is the most relevant factor, both in its linear and quadratic form. Moreover, the results revealed that the interaction between pH and temperature exhibited a significant effect on protein extraction. Extraction time and the meal/water ratio did not seem to affect the protein purity in the selected range. Response surface methodology suggested that the highest protein content of GSPC could be achieved at pH 10, at a temperature of 36 °C, with a meal/water ratio of 1:9 and an extraction time of 2 h (54.50%). The optimized conditions resulted in an experimental protein purity of 55.35%. The agreement between the predicted and experimental values showed the suitability of the model equation for predicting the optimum response value. The removal of phenolic compounds from grape seed meal, before optimizing protein extraction, led to a significant increase in the protein purity of grape seed protein concentrate, and reached 59.6%. The protein content in OGSPC justifies its use as a sustainable means of protein production in the oenological sector due to the availability of grape seeds and their low cost as a grape-processing by-product. However, further studies on the increase in the protein purity of the isolates are needed to be explored. A comprehensive study should be made to evaluate other factors and other ranges of the studied factors that have not been significant in this study.

## Figures and Tables

**Table 1:** Independent variables and their levels in the response surface design.

Independent Variables	Coded Symbols	Coded Factor Levels		
		−1	0	1
pH	x_1_	8.5	9.5	10.5
Temperature (°C)	x_2_	25	35	45
Meal/water ratio (*w*/*v*)	x_3_	1:6	1:9	1:12
Extraction time (h)	x_4_	1	2	3

**Table 2:** Box–Behnken design (BBD) and responses (experimental and predicted protein contents) for the optimization of the protein concentrate extraction of defatted grape seed meal.

Coded Variables				Uncoded Variables				Protein Content (%)	
x_1_ (pH)	x_2_ (T)	x_3_ (m/w)	x_4_ (time)	x_1_ (pH)	x_2_ (T)	x_3_ (m/w)	x_4_ (time)	Experimental ^1^	Predicted
−1	−1	0	0	8.5	25	1:9	2	42.87 ± 3.16 ^bc^	38.23
−1	0	−1	0	8.5	35	1:6	2	44.89 ± 6.91 ^bcd^	45.36
−1	0	0	−1	8.5	35	1:9	1	43.06 ± 1.32 ^bc^	44.18
−1	0	0	1	8.5	35	1:9	3	38.71 ± 2.06 ^ab^	41.49
−1	0	1	0	8.5	35	1:12	2	34.81 ± 1.20 ^a^	39.72
−1	1	0	0	8.5	45	1:9	2	57.93 ± 0.63 ^hi^	53.29
0	−1	−1	0	9.5	25	1:6	2	54.15 ± 1.35 ^efghi^	55.09
0	−1	0	−1	9.5	25	1:9	1	54.71 ± 4.68 ^efghi^	55.34
0	−1	0	1	9.5	25	1:9	3	53.45 ± 1.03 ^efghi^	53.79
0	−1	1	0	9.5	25	1:12	2	53.05 ± 0.43 ^efghi^	52.45
0	0	−1	−1	9.5	35	1:6	1	50.83 ± 1.14 ^defg^	51.34
0	0	−1	1	9.5	35	1:6	3	53.22 ± 0.55 ^efghi^	52.82
0	0	0	0	9.5	35	1:9	2	47.94 ± 1.27 ^cde^	53.34
0	0	0	0	9.5	35	1:9	2	56.88 ± 1.43 ^ghi^	53.34
0	0	0	0	9.5	35	1:9	2	55.19 ± 2.25 ^fghi^	53.34
0	0	1	−1	9.5	35	1:12	1	53.38 ± 0.71 ^efghi^	52.93
0	0	1	1	9.5	35	1:12	3	53.30 ± 1.95 ^efghi^	51.03
0	1	−1	0	9.5	45	1:6	2	52.84 ± 1.10 ^efghi^	54.91
0	1	0	−1	9.5	45	1:9	1	58.86 ± 0.93 ^i^	57.79
0	1	0	1	9.5	45	1:9	3	59.13 ± 0.82 ^i^	57.77
0	1	1	0	9.5	45	1:12	2	55.70 ± 1.39 ^ghi^	57.36
1	−1	0	0	10.5	25	1:9	2	56.97 ± 1.17 ^ghi^	60.31
1	0	−1	0	10.5	35	1:6	2	51.80 ± 1.23 ^efgh^	48.20
1	0	0	−1	10.5	35	1:9	1	53.25 ± 0.27 ^efghi^	52.51
1	0	0	1	10.5	35	1:9	3	52.73 ± 1.40 ^efghi^	53.64
1	0	1	0	10.5	35	1:12	2	56.89 ± 3.26 ^ghi^	53.65
1	1	0	0	10.5	45	1:9	2	48.34 ± 1.14 ^cdef^	51.68

^1^ Mean of triplicate measurements; means values with different letters in the same column indicate statistical differences (Tukey test, α = 0.05).

**Table 3:** ANOVA and regression coefficients of the second-order polynomial model for the response variable (protein purity).

Source	DF	Coefficients	Sum of Squares	Mean Squares	F-Value	*p*-Value
Intercept		−713.726				
Linear						
x_1_ (pH)	1	132.810	185.3846	185.3846	11.95148	0.004743
x_2_ (T)	1	4.034	14.7821	14.7821	0.95298	0.348231
x_3_ (water/meal ratio)	1	907.545	0.0299	0.0299	0.00193	0.965698
x_4_ (extraction time)	1	−12.887	0.1229	0.1229	0.00792	0.930552
Quadratic						
x_1_^2^	1	−5.341	152.1330	152.1330	9.8078	0.008664
x_2_^2^	1	0.029	44.2030	44.2030	2.8497	0.117186
x_3_^2^	1	−830.218	8.3426	8.3426	0.53783	0.477416
x_4_^2^	1	−0.043	0.0101	0.0101	0.00065	0.980106
Interaction						
x_1 × 2_	1	−0.592	140.3019	140.3019	9.04507	0.010913
x_1 × 3_	1	−66.581	33.0650	33.0650	2.13165	0.169965
x_1 × 4_	1	0.955	3.6492	3.6492	0.23526	0.636385
x_2 × 3_	1	−3.053	6.9524	6.9524	0.44821	0.515861
x_2 × 4_	1	0.038	0.5887	0.5887	0.03795	0.848803
x_3 × 4_	1	20.290	3.0708	3.0708	0.19797	0.664285
Residual	12	-	186.1372	15.5114	-	-
Lack of fit	10	-	141.0803	14.1080	0.626233	0.749869
Pure error	2	-	45.0568	22.5384	-	-
Total	26	-	966.5705	-	-	-
*R* ^2^	0.80743					
*R*^2^ (adjusted)	0.58275					
CV (%)	4.073					

**Table 4:** The amino acid composition of grape seed protein concentrate (GSPC) (g aa/100 g protein and %).

Amino Acid	Grape Seed Protein Concentrate ^1^ (GSPC)	
	g aa/100 g Protein	%
Asp	7.18 ± 0.28	8.42
Thr	1.77 ± 0.06	2.07
Ser	3.35 ± 0.14	3.93
Glu	27.93 ± 0.98	32.76
Gly	7.96 ± 0.29	9.34
Ala	3.23 ± 0.14	3.79
Cus	0.80 ± 0.02	0.94
Val	3.67 ±0.15	4.30
Met	1.19 ± 0.05	1.40
Ile	2.93 ± 0.11	3.43
Leu	4.73 ± 0.25	5.55
Tyr	2.65 ± 0.08	3.11
Trp	-	-
Phe	3.89 ± 0.15	4.56
His	1.81 ± 0.02	2.13
Lys	1.98 ± 0.04	2.33
Arg	7.29 ± 0.27	8.55
Pro	2.88 ± 0.20	3.38

^1^ Mean of triplicate measurements.

**Table 5:** Protein content (%) of the optimized grape seed protein concentrate (GSPC) with and without phenols and mucilage.

Sample	Extraction Conditions				Protein Content ^1^ (%)
	pH	T (°C)	w:m (*w*/*v*)	Time (h)	
Grape seed protein concentrate (GSPC)	10	36	9:1	2	55.35 ± 0.10 ^a^
Grape seed protein concentrate without phenols	10	36	9:1	2	59.6 ± 2.48 ^b^
Grape seed protein concentrate without mucilage	10	36	9:1	2	58.06 ± 0.39 ^ab^

^1^ Mean of triplicate experiments; means values with different letters in the same column indicate statistical differences (Tukey test, α = 0.05).

## Data Availability

Data is contained within the article.

## Abbreviations

RSM, response surface methodology; BBD, Box–Behnken design; PC, protein concentrate; GSPC, grape seed protein concentrate; OGSPC, optimized grape seed protein concentrate; ANOVA, analysis of variance.
